# Evaluation of Secretion Prediction Highlights Differing Approaches Needed for Oomycete and Fungal Effectors

**DOI:** 10.3389/fpls.2015.01168

**Published:** 2015-12-23

**Authors:** Jana Sperschneider, Angela H. Williams, James K. Hane, Karam B. Singh, Jennifer M. Taylor

**Affiliations:** ^1^CSIRO Agriculture Flagship, Centre for Environment and Life SciencesPerth, WA, Australia; ^2^The Institute of Agriculture, The University of Western AustraliaCrawley, WA, Australia; ^3^Department of Environment and Agriculture, CCDM Bioinformatics, Centre for Crop and Disease Management, Curtin UniversityPerth, WA, Australia; ^4^Curtin Institute for Computation, Curtin UniversityPerth, WA, Australia; ^5^CSIRO Agriculture, Black Mountain LaboratoriesCanberra, ACT, Australia

**Keywords:** signal peptide prediction, effectors, protein secretion, fungi, oomycetes, plant pathogens

## Abstract

The steadily increasing number of sequenced fungal and oomycete genomes has enabled detailed studies of how these eukaryotic microbes infect plants and cause devastating losses in food crops. During infection, fungal and oomycete pathogens secrete effector molecules which manipulate host plant cell processes to the pathogen's advantage. Proteinaceous effectors are synthesized intracellularly and must be externalized to interact with host cells. Computational prediction of secreted proteins from genomic sequences is an important technique to narrow down the candidate effector repertoire for subsequent experimental validation. In this study, we benchmark secretion prediction tools on experimentally validated fungal and oomycete effectors. We observe that for a set of fungal SwissProt protein sequences, SignalP 4 and the neural network predictors of SignalP 3 (*D*-score) and SignalP 2 perform best. For effector prediction in particular, the use of a sensitive method can be desirable to obtain the most complete candidate effector set. We show that the neural network predictors of SignalP 2 and 3, as well as TargetP were the most sensitive tools for fungal effector secretion prediction, whereas the hidden Markov model predictors of SignalP 2 and 3 were the most sensitive tools for oomycete effectors. Thus, previous versions of SignalP retain value for oomycete effector prediction, as the current version, SignalP 4, was unable to reliably predict the signal peptide of the oomycete Crinkler effectors in the test set. Our assessment of subcellular localization predictors shows that cytoplasmic effectors are often predicted as not extracellular. This limits the reliability of secretion predictions that depend on these tools. We present our assessment with a view to informing future pathogenomics studies and suggest revised pipelines for secretion prediction to obtain optimal effector predictions in fungi and oomycetes.

## Introduction

The growing number of sequenced fungal and oomycete plant pathogen genomes has enabled detailed reverse genetics studies into molecular pathogen-host interactions (Dean et al., 2012; Kamoun et al., 2014). Though fungi and oomycetes belong to phylogenetically distinct microbial taxa, they both use a diverse class of molecules, termed effectors, to promote pathogenicity through subversion of host defenses or impairment of normal host-cell function (Dodds and Rathjen, 2010; Lo Presti et al., 2015). Effector molecules may be the products of either secondary metabolite or protein synthesis, however the majority of effectors identified in fungi and oomycetes are the latter. Proteinaceous effectors are initially synthesized intracellularly and require relocation to the extracellular space (apoplastic effectors) or subsequent import into the host cell cytoplasm or specific organelles (cytoplasmic effectors). The classical endoplasmic reticulum (ER)/Golgi-dependent secretion pathway in eukaryotes is well-defined and involves recognition of an N-terminal signal peptide that is cleaved off as the protein is translocated across the membrane (Von Heijne, 1990). Classical signal peptides can be predicted computationally with high accuracy (Menne et al., 2000; Klee and Ellis, 2005; Choo et al., 2009; Min, 2010; Melhem et al., 2013), and the majority of experimentally verified fungal and oomycete effectors are predicted to be secreted in this manner. However, reports are emerging for yet unknown, non-classical secretion pathways to also play a role in fungal and oomycete effector externalization (Ridout et al., 2006; Liu et al., 2014). Numerous eukaryotic plant pathogen effectors have been found to be active inside the host cell cytoplasm; however the knowledge of how effectors are delivered into the plant cells after secretion is fragmentary. In oomycete effectors, conserved amino acid motifs such as RXLR, CHXC, or LFLAK are positioned in N-terminal domains and define oomycete effector superfamilies (Petre and Kamoun, 2014). Although mechanisms have been proposed as to how the RXLR motif may facilitate cell entry through the host cell membrane phospholipid bilayer, the results are still controversial (Tyler et al., 2013; Wawra et al., 2013). Conserved sequence motifs associated with translocation have thus far not been found for fungal effector proteins, which makes their computational prediction from secretomes challenging (Sperschneider et al., 2015). A conserved Y/F/WxC-motif has been identified in the N-terminus of effector candidates in the barley powdery mildew fungus (Godfrey et al., 2010), however the role of this motif in cell entry or pathogenicity remains undetermined.

Several studies have exploited proteomics to experimentally identify secreted proteins involved in pathogenicity. For example, an early proteomics study of extracellular proteins of the wheat-infecting fungus *Fusarium graminearum* identified 120 candidates secreted *in planta*, of which only 56% possessed a predicted signal peptide motif (Paper et al., 2007). A later study in the same species identified only 69 secreted proteins, following growth in barley or wheat flour-based liquid cultures to mimic host-pathogen interactions (Yang et al., 2012). Of these, 70% possessed a predicted signal peptide. A recent study in the oomycete potato pathogen *Phytophthora infestans* predicted 80% of its extracellular proteome to contain a signal peptide (Meijer et al., 2014). Thus, there appears to be wide variability (both between species and experiments) in the number of extracellular proteins identified through experimental proteomics that are also predicted to be secreted *in silico*. A high percentage of proteins lacking a classical signal peptide may be due to contamination of extracellular samples with intracellular proteins, due to rupture of the fungal cells during the protein extraction procedure. Furthermore, protein extraction may be complicated in species where there is a low or variable pathogen biomass relative to the host, or that selectively secrete different proteins when grown in different *in vitro* or infection-mimicking cultures. Computational limitations also have the potential to complicate proteomics experiments. This may come from variability between species in their use of non-classical secretion mechanisms, which cannot yet be accurately predicted. Gene annotation is also an important determining factor for the reliability of both experimental proteomics and computational prediction of secretion. Proteomic identification of genes is dependent on the completeness and accuracy of translated gene annotations that are used to generate a searchable database of predicted trypsin-digested proteins, to which peptide mass-spectra are matched (Bringans et al., 2009). Thus, missing or incorrect gene annotations may exclude or confuse identification of extracellular proteins. Prediction of secretion also relies strongly on the presence and accurate annotation of the 5′ exons of genes, which encode N-terminal signal peptides. Due to these technical difficulties, deriving accurate computational predictions of secreted proteins from whole genome sequences remains an important pursuit in plant pathology, with a view toward efficient identification of secreted proteins for subsequent effector prediction.

The apparent ease of secretion prediction has led to its common use in pathogenomic studies as a first pass filter in narrowing down a whole proteome dataset into a short-list of potential effector candidates (Kämper et al., 2006; Raffaele et al., 2010; Rouxel et al., 2011; Hane et al., 2014; Nemri et al., 2014). A variety of software tools exists for eukaryotes that can predict whether proteins are secreted into the extracellular environment (Emanuelsson et al., 2007). Typically, this involves recognition of the N-terminal secretory signal peptide motif that directs proteins through the classical ER/Golgi-dependent pathway using tools such as SignalP (Petersen et al., 2011). Whilst this is a robust approach for defining a set of potential effector candidates, typically far more candidates are predicted for experimental validation than is feasible. Furthermore, proteins that are predicted to be secreted via a classical pathway might be retained in the ER/Golgi or fulfill roles as part of the cell wall. Therefore, subcellular localization prediction is an important tool that can point toward the functional role or interaction partners of a protein based on its amino acid sequence and can be used to assess if a protein is indeed secreted into the extracellular space (Emanuelsson, 2002). Transmembrane proteins are also commonly predicted and removed from the secretome as these are likely to fulfill functions in the pathogen cell wall. Whilst *in silico* methods for secretome prediction are under active development and show robust performance, their reported predictive accuracy strongly depends on the selection of the test set and independent benchmarking studies are important for an unbiased tool evaluation. For example, a comprehensive benchmark of secretion prediction tools found that predictive accuracy was in many cases lower than those initially reported by the developers (Klee and Ellis, 2005). Although an evaluation on a large test set covering a wide taxonomic spectrum gives a good indication of a tool's performance, it provides limited insight into its expected performance on a specialized set of proteins, such as effector proteins of fungal and oomycete pathogens.

This study set out to reveal the strengths and weaknesses of existing protein secretion and subcellular localization prediction methods, as applied to the identification of effector proteins produced by fungi or oomycete plant pathogens. Prediction pipelines that have been used in previous studies for defining secretomes and subsequently effector candidates of eukaryotic plant pathogens are diverse and highly parameterized, as exemplified in Table 1. For example, SignalP (Nielsen et al., 1997; Nielsen and Krogh, 1998; Bendtsen et al., 2004b; Petersen et al., 2011) or Phobius (Käll et al., 2004) are utilized by the majority of pipelines to extract proteins that are likely to be secreted via a classical pathway. Despite the availability of the latest version of SignalP 4, which was designed to discriminate between signal peptides and N-terminal transmembrane (TM) regions, previous versions (2 and 3) are still frequently used due to their increased sensitivity. Phobius was designed to predict secretion and N-terminal TM domains separately, predicting both the presence of a signal peptide and the number and location of TM helices.

**Table 1:** **Examples for approaches used in eukaryotic plant pathogen genomic studies that predict secreted proteins**.

**References**	**Species/taxon**	**Secretome size (total number of predicted genes)**	**Positive evidence**	**Negative evidence**	**Remarks**
**(A) FUNGAL PATHOGENS**					
Kämper et al., 2006	*Ustilago maydis*	426 (6902)	SignalP 3.0, TargetP, ProtComp 6.0		
Ma et al., 2010	*Fusarium oxysporum* f. sp. *lycopersici*	1803 (17,735)	SignalP 3.0	TMHMM, Phobius, TargetP	SignalP *D*-score, TM allowed in the first 50 amino acids, TargetP no mitochondrial targeting with RC = 1 or 2
	*F. verticillioides*	1549 (14,179)			
Spanu et al., 2010	*Blumeria graminis* f. sp. *hordei*	NA (5854)	SignalP, SecretomeP	–	*S*-prob > 0.9, SecretomeP > 0.5
Rouxel et al., 2011	*Leptosphaeria maculans*	NA (12,469)	SignalP 3.0, TargetP	TMHMM	Both SignalP-NN and SignalP-HMM predict SP, TargetP predicts “secreted,” 1 TM in SP allowed
Duplessis et al., 2011	*Melampsora larici*-*populina*	NA (16,399)	SignalP	TargetP, TMHMM, big-PI	*S*-prob > 0.9, TargetP no mitochondrial targeting with RC = 1 or 2, one TM ≥ 18 in first 60 aas allowed
	*Puccinia graminis* f. sp. *tritici*	NA (17,773)			
Klosterman et al., 2011	*Verticillium dahliae*	780 (10,535)	SignalP 3.0, WoLF PSORT	TMHMM, Phobius	*D*-score > 0.5, WoLF PSORT predicted to be extracellular, no TM predicted by either TMHMM or Phobius
	*V. albo-atrum*	759 (10,221)			
Cantu et al., 2011	*P. striiformis* f. sp. *tritici*	1088 (20,423)	SignalP 3.0	TMHMM, TargetP	*S*-prob ≥ 0.9, average cleavage site position: 24 ± 9, TM length ≥ 18 aas and not in the first 60 aas, TargetP no mitochondrial proteins (RC = 1 or 2)
O'Connell et al., 2012	*Colletotrichum higginsianum*	2142 (16,172)	WoLF PSORT	–	WoLF PSORT predicted to be extracellular
	*C. graminicola*	1650 (12,006)			
Ohm et al., 2012	Dothideomycetes	NA	SignalP 3.0	TMHMM 2.0	One TM allowed in first 40 aas
Brown et al., 2012	*Fusarium graminearum*	574 (13,937)	SignalP 3.0, TargetP 1.1, ProtComp 8.0, WoLF PSORT 0.2	TMHMM 2.0, big-PI	SignalP *D*-score = Y, TargetP predicts “secreted,” ProtComp extracellular or unknown, no TM in mature protein, WoLF PSORT extracellular score > 17, no GPI-anchor
de Wit et al., 2012	*Cladosporium fulvum*	1200 (14,127)	SignalP 3.0, WoLF PSORT	Phobius, TMHMM 2.0, TargetP 1.1, PredGPI	*D*-score > 0.5, TargetP no mitochondrial targeting, no GPI anchor, no TM predicted by either TMHMM or Phobius
	*Dothistroma septosporum*	905 (12,580)			
Wiemann et al., 2013	*Fusarium fujikuroi*	1336 (14,813)	SignalP 4.0, SecretomeP, WoLF PSORT	TargetP, TMHMM	SecretomeP score > 0.6, TargetP no mitochondrial targeting, TargetP RC-score < 4, Signalp *S*-score > 0.5
Manning et al., 2013	*Pyrenophora tritici-repentis*	1146 (12,141)	SignalP 3.0, WoLF PSORT	TMHMM	WoLF PSORT predicted to be extracellular, one TM allowed unless it starts in the first 10 aas
Hane et al., 2014	*Rhizoctonia solani* AG8	1959 (13,964)	SignalP 4.1, Phobius 1.01, WoLF PSORT 0.2	Phobius 1.01	SignalP secreted or Phobius secreted or WoLF PSORT predicted to be extracellular, only one TM domain allowed
Nemri et al., 2014	*Melampsora lini*	1085 (26,443)	SignalP 2.1-HMM, SignalP 4.1	TMHMM 2.0, TargetP 1.1	No TM, TargetP no mitochondrial targeting, *D*-score > 0.36, S-prob > 0.88, cleavage site predicted between amino-acid 10 and 40 using SignalP 2
Guyon et al., 2014	*Sclerotinia sclerotiorum*	745 (14,503)	SignalP 2, SignalP 4	TMHMM, GPIsom	TM after SP removed, no GPI anchor
**(B) OOMYCETE PATHOGENS**					
Haas et al., 2009	*Phytophthora infestans*	NA (17,797)	SignalP 3.0	–	–
Raffaele et al., 2010	*P. infestans*	1415 (18,155)	SignalP 2.0, SignalP 3.0, TargetP, PSort	TMHMM	SignalP-HMM 2.0 score ≥ 0.9, SignalP-NN 3.0 *Y-max* score ≥ 0.5, SignalP- NN 3.0 *D*-score ≥ 0.5, SignalP- HMM 3.0 *S*-prob ≥ 0.9, TargetP predicted localization “Secreted” (S), most probable PSort location “extracellular” (extr.) and no TMHMM predicted TM domain after signal peptide cleavage site
Lévesque et al., 2010	*Pythium ultimum*	747 (15,297)	SignalP 2.0	TMHMM, TargetP	SignalP-HMM predicts signal peptide, SignalP-NN predicts a cleavage site between amino acids 10 and 40
Links et al., 2011	*Albugo candida*	939 (15,824)	SignalP 3.0	–	Either SignalP-NN or SignalP-HMM predict SP, SignalP predicts a cleavage site between amino acids 10 and 30
Kemen et al., 2011	*A. laibachii*	1636 (13,032)	SignalP 3.0	MEMSAT3	Both neural network and hidden Markov model predict signal peptide. Proteins were considered to be without a TM domain with *p*_non−TM_> 0.0004 or, for high stringency, *p*_non−TM_> 0.01.
**(C) SECRETOME STUDIES**					
Lowe and Howlett, 2012	Fungi	–	SignalP	–	–
Sperschneider et al., 2015	Fungi	–	SignalP 4.1	–	–
Lo Presti et al., 2015	Fungi	–	SignalP 4.0	TMHMM	No TMs as predicted by TMHMM 2.0c (TMHMM score < 2)

*If provided in the original paper, version numbers of prediction tools are given*.

Furthermore, there are also discrepancies in how tools are used and how thresholds for secretion are set (Table 1). For example, some studies have used the neural network scores from SignalP 2 and 3 with custom thresholds, whereas others rely on the hidden Markov model probability for predicting the presence of a signal peptide. SignalP 2 and 3 employ predictions from both a neural network (SignalP-NN) and a hidden Markov model (SignalP-HMM), whilst the latest version SignalP 4 is purely based on neural networks. SignalP 2 returns three neural network scores for each position in the sequence: a raw cleavage site score (*C*-score), the signal peptide score (*S*-score), and the combined cleavage site score (*Y*-score). For each sequence, it reports the maximal *C*-, *S*-, and *Y*-scores as well as the mean *S*-score between the N-terminus and the predicted cleavage site that it used to assess whether a sequence contains a signal peptide. Furthermore, it returns two hidden Markov model scores, the *C*-score as well as the probability that the sequence contains a signal peptide (*S*-probability). SignalP 3 replaces the previously used mean *S*-score for classification with the *D*-score, which is calculated as the average of the mean *S*-score, and the maximal *Y*-score. It still uses both neural network scores and calculates the signal peptide probability with a hidden Markov model. SignalP 4 is a neural network based method designed to discriminate between signal peptides and transmembrane regions. Prediction of signal peptides is based entirely on the *D*-score. For all scores, Boolean flags are provided which are either “Y” for a signal peptide or “N” for no signal peptide.

Subcellular localization tools such as TargetP (Emanuelsson et al., 2000), WoLF PSORT (Horton et al., 2007), or ProtComp are frequently used to complement the predictions made by SignalP or Phobius, either through a union or intersection of predictions made by these methods (Table 1). This can serve to filter proteins that may be predicted to contain a signal peptide, yet that might not be fully secreted into the extracellular space due to being retained within the ER/Golgi. TargetP predicts if a protein is secreted or localized to the mitochondria, chloroplast, or another unknown location. It reports reliability class scores from 1 to 5, where 1 corresponds to the strongest prediction. Another tool WoLF PSORT, an updated version of PSORT II, has been trained separately on fungi, animal, and plant data. It reports predicted subcellular locations (nuclear, mitochondria, cytosol, cytoskeleton, endoplasmic reticulum, plasma membrane, extracellular, chloroplast, peroxisome, Golgi apparatus, lysosome, and vacuolar membrane) in terms of respective scores based on a weighted *k*-nearest neighbor classifier. The output format is similar to a sequence similarity search, with scores assigned for each predicted localization site based on the number of nearest neighbors to the query protein. In most studies that employ WoLF PSORT, proteins have been predicted as secreted where extracellular predictions score higher than other locations (Table 1). Less commonly, the prediction of non-classically secreted proteins has been reported using SecretomeP, which has been trained on a very small set of verified non-classically secreted proteins derived from mammalian and bacterial sequences (Bendtsen et al., 2004a). Consequently, the relevance of SecretomeP to fungal and oomycete proteins is questionable. Finally, ProtComp is a web-server based tool combining several methods for protein localization, ranging from neural networks to sequence homology searches. Its lack of a publicly distributed version for local installation precludes it from routine use for whole-genome analysis. Predicted transmembrane proteins are typically removed from the set of predicted extracellular proteins using programs such as TMHMM (Krogh et al., 2001) or Phobius. However, most pipelines allow for the presence of one transmembrane domain in the N-terminus, as this can correspond to the signal peptide as both are predicted based on the presence of hydrophobic residues. Additionally, TargetP is often employed to eliminate proteins predicted to be targeted to mitochondria or chloroplasts. In some fungal studies, predicted GPI-anchored proteins are also removed from the set of secreted effector candidates.

The diversity of prediction pipelines shown in Table 1 illustrates an overall lack of consensus used to predict extracellular pathogen proteins, in particular for effector candidates, and presents difficulties when comparing secretome sizes across different species. Herein we benchmark the performance of individual secretion prediction tools on experimentally verified fungal and oomycete effectors and use the best-performing tools to predict extracellular proteins across fungal and oomycete pathogens. In particular, we show that for cytoplasmic effector proteins that are first secreted into the extracellular space and subsequently translocated to the host cell, protein subcellular localization predictors suffer from poor accuracy. We highlight differences in performance for secretion prediction between fungal effectors and oomycete effectors and conclude by providing practical recommendations for the computational secretion prediction for effector candidate mining from eukaryotic pathogen genomes.

## Materials and methods

Various datasets were chosen for the purpose of comparing the performance of secretion prediction software tools, in the context of plant pathogenomics. Experimentally validated fungal and oomycete effector protein sequences were collected from PHIbase version 3.6 (Urban et al., 2015) and from manual literature searches (Supplementary Data Sheet 1, 2, Supplementary Table 1). For further benchmarking, representative datasets for both extracellular and intracellular proteins of the fungi were obtained by searching SwissProt database records created between 2011 and 2015 for: (1) fungal proteins that have been manually annotated as secreted (taxonomy:“Fungi [4751]” locations:(location:“Secreted [SL-0243]” evidence:manual) created:[20110101 TO 20150101]) (Supplementary Data Sheet 3); and (2) fungal proteins that have been manually annotated as localized to the nucleus (taxonomy:“Fungi [4751]” locations:(location:“Nucleus [SL-0191]” evidence:manual) created:[20110101 TO 20150101]) (Supplementary Data Sheet 4). Sequences that did not start with “M” or were shorter than 30 aas were removed. Both sets only cover proteins for which entries were created after 2011, to avoid an overlap with the training sets used for secretion prediction tools. We could not extract an equivalent set for oomycete proteins from SwissProt due to the very low number of entries for manually curated secreted proteins (four entries). Secretion prediction tools were run on a local machine, or using web servers where indicated, as in Table 2 (Results given in Supplementary Data Sheet 5). Sensitivity was calculated as *TP*/(*TP* + *FN*) and specificity as *TN*/(*TN* + *FP*), where *TP* is the number of true positives, *TN* the number of true negatives, *FP* the number of false positives and *FN* the number of false negatives. The Matthews correlation coefficient (MCC) was calculated as $\frac{TP\times TN-FP\times FN}{\sqrt{\left(TP+FP\right)\left(TP+FN\right)\left(TN+FP\right)\left(TN+FN\right)}}$.

**Table 2:** **Software tested in this study and the parameters under which proteins were predicted to be secreted**.

**Software**	**References**	**Secretion or transmembrane prediction criteria**
SignalP 2.0	Nielsen et al., 1997 Nielsen and Krogh, 1998	SignalP-HMM: labeled “Y” based on *S*-probability (default threshold 0.5). SignalP-NN: labeled “Y” based on *S*-score (default threshold 0.47).
SignalP 3.0	Bendtsen et al., 2004b	SignalP-HMM: labeled “Y” based on *S*-probability (default threshold 0.5). SignalP-NN: labeled “Y” based on *D*-score (default threshold 0.43).
SignalP 4.1	Petersen et al., 2011	Labeled “Y” based on *D*-score (default thresholds 0.45 for SignalP-noTM networks and 0.5 for SignalP-TM networks).
Phobius	Käll et al., 2004	Predicted as secreted if presence of a signal peptide (SP) labeled as “Y.” Transmembrane protein if the number of predicted transmembrane segments (TM) is ≥ 1.
TargetP 1.1	Emanuelsson et al., 2000	Labeled “S” for signal peptide (“secreted”), regardless of the reliability class score.
WoLF PSORT 0.2	Horton et al., 2007	Secreted if the best score in the ranked localization list is “extracellular.”
ProtComp 9.0^*^	http://www.softberry.com/berry.phtml	Secreted if the integral prediction of protein location contains “extracellular (secreted).”
TMHMM 2.0	Krogh et al., 2001	Transmembrane protein if one or more transmembrane helices beginning outside the first 60 aas.

*All tools were run with default parameters and settings*.

* *Run using web-server*.

## Results and discussion

### Signalp 2, 3 and 4 show the best performance for secretion prediction on a set of fungal protein sequences

Several independent benchmark analyses have been published that compare the accuracy of secretion prediction tools. For example, Klee and Ellis (2005) evaluated a range of secretion prediction methods (SignalP 3.0, SignalP 2.0, TargetP 1.01, PrediSi, Phobius, and ProtComp 6.0) on 372 proteins from five vertebrate organisms and found that TargetP, the SignalP 3 maximum *S*-score and SignalP 3 *D*-score were the most accurate single scores. Choo et al. (2009) found that most of the tested tools were capable of reliably distinguishing secreted from non-secreted proteins, as indicated by the high specificities that were achieved. SignalP 4 has been reported by the authors to outperform previous versions of SignalP for a test set spanning eukaryotic and bacterial sequences (Petersen et al., 2011).

Min (2010) evaluated eukaryotic secretion prediction using Phobius, SignalP 3.0, TargetP, and WoLF PSORT individually and in combination with TMHMM and PS-Scan and found that for fungi the most reliable individual predictor of secretion was WoLF PSORT, but a combination of tools produced the most accurate predictions. A follow-up study including SignalP 4.0 reported WoLF PSORT as the best individual tool for fungal data and also made the general recommendation of using SignalP 4.0 over SignalP 3.0 (Melhem et al., 2013). However, the authors assign a protein as predicted to be secreted by WoLF PSORT if it features “extracellular” in the ranked localization list whereas other studies (Table 1), including ours, have used this tool quite differently requiring more stringently that the “extracellular” score is higher than that of all other sub-cellular locations. Notably, WoLF PSORT stands out amongst the tools compared in that it has been trained on a relatively extensive set of fungal proteins. However, while it performs well for fungal secreted proteins overall, when restricted to known secreted effectors its performance is markedly poorer.

For the evaluation of secretion prediction performance we utilized two data sets from the SwissProt database: one that contained fungal proteins that were manually annotated as secreted (409 proteins) and the other that contained non-secreted fungal proteins that were manually annotated as nuclear (1113 proteins). We could not extract an equivalent set for oomycete proteins from SwissProt due to the very low number of entries for manually curated secreted or nuclear proteins. All tools tested achieved high specificity in the range of 97.2–99.8%, whereas sensitivity varied more dramatically (Table 3). All versions of SignalP, Phobius, and TargetP achieved high sensitivity of more than 94.9%. In contrast, the proportion of proteins that are predicted to be extracellular by WoLF PSORT and ProtComp showed lower sensitivity at 88 and 63.3%, respectively. In terms of the Matthews correlation coefficient (MCC), SignalP 4, SignalP-NN 3 (*D*-score), and SignalP-NN 2 perform best (MCC = 0.96), with SignalP 2 and 3 showing slightly more sensitivity than SignalP 4, which in turn achieves marginally higher specificity. These results confirm the strong predictive performance of SignalP for secreted fungal proteins.

**Table 3:** **Performance of secretion prediction tools applied to secreted fungal proteins sourced from SwissProt**.

**Measure**	**SignalP 4.1**	**SignalP-NN 3.0 (*D*-Score)**	**SignalP-HMM 3.0**	**SignalP-NN 2.0**	**Signal-HMM 2.0**	**Phobius**	**TargetP 1.1**	**WoLF PSORT**	**ProtComp 9.0**
Sensitivity	94.9%	95.6%	95.8%	95.6%	95.8%	95.4%	95.1%	88%	63.3%
Specificity	99.7%	99.6%	98.4%	99.4%	98.2%	98.7%	98.3%	99.8%	97.2%
MCC	**0.96**	**0.96**	0.94	**0.96**	0.94	0.94	0.93	0.91	0.68

*Sensitivity, specificity and the Matthews correlation coefficient (MCC) are shown for evaluating the performance of secretion prediction tools. All tools were run with the settings and parameters given in Table 2. The best performance in terms of MCC is marked in bold*.

### Differences in sensitivity of secretion prediction tools for effectors from fungi and oomycetes

**Figure 1:**
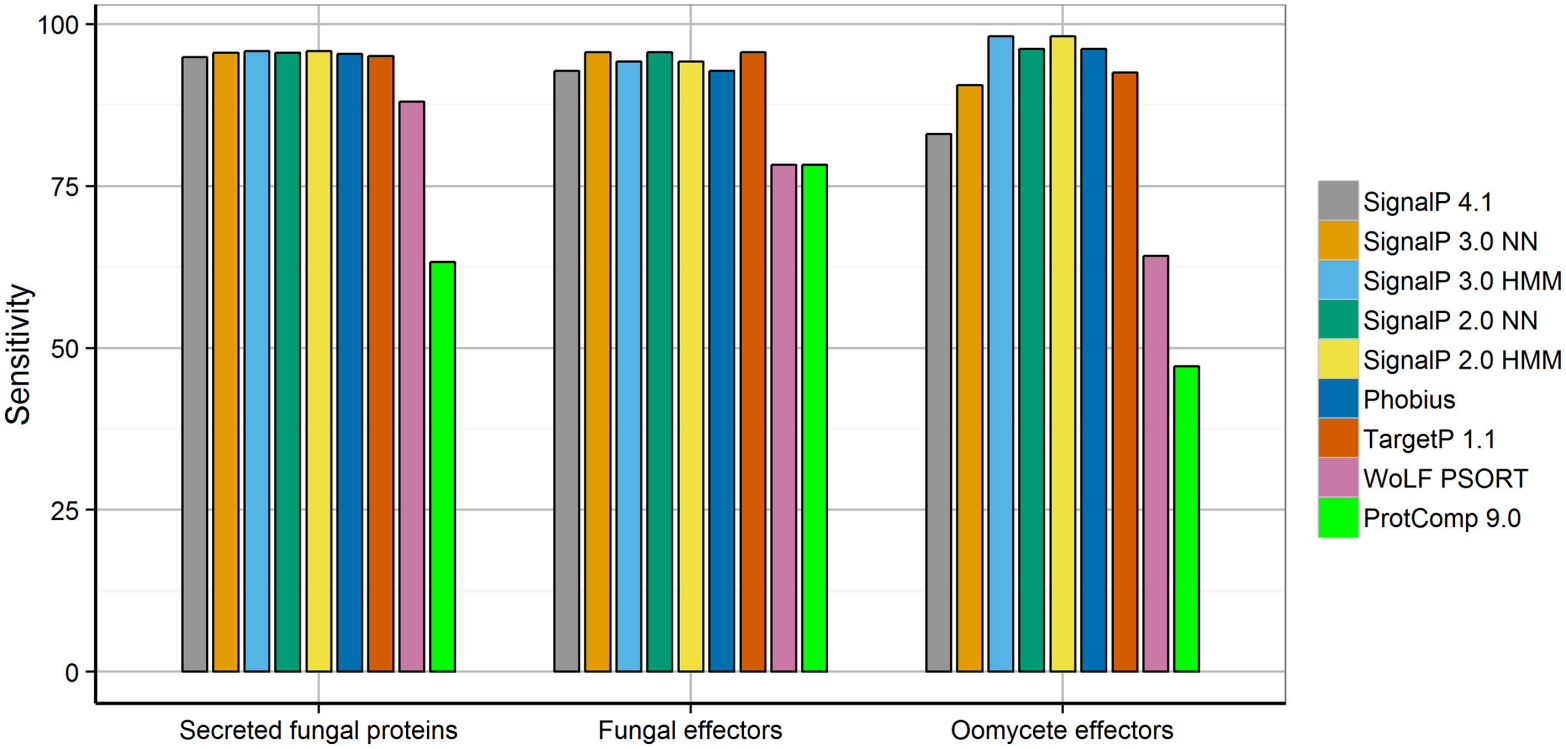
**Sensitivity of secretion prediction tools for secreted fungal proteins, fungal effectors, and oomycete effectors**. Differences in secretion prediction sensitivity are shown for the set of secreted fungal proteins taken from SwissProt as well as the sets of experimentally verified fungal and oomycete effectors.

In line with previous studies (Menne et al., 2000; Klee and Ellis, 2005; Choo et al., 2009; Min, 2010), we found that all tools tested achieved high specificity in secretion prediction. For effector prediction in particular, the use of a sensitive method can be desirable to obtain the most complete candidate effector set. To test the sensitivity of secretion prediction tools for effector proteins from eukaryotic plant pathogens, we collected two sets of experimentally verified fungal and oomycete effectors from the literature. In total, the test set of fungal and oomycete effectors contain 69 and 53 proteins, respectively, (Supplementary Table 1). Interestingly, the sensitivity of secretion prediction tools varied between the fungal and oomycete effector sets (Figure 1). The neural network predictors of SignalP 3 and SignalP 2 (SignalP-NN 2, SignalP-NN 3) as well as TargetP (“S” for secreted with RC scores ranging from 1 to 5) were found to be the most sensitive for fungal effectors (95.7%). In contrast, the hidden Markov model predictors of SignalP 2 and SignalP 3 (SignalP-HMM 2, SignalP-HMM 3) achieved highest sensitivity for oomycete effectors (98.1%). In general, neural networks and hidden Markov models have different strengths in pattern recognition tasks. Whereas neural networks are powerful for correlating features over a longer range, hidden Markov models are advantageous for modeling sequential regions or patterns found in signal peptides (Nielsen et al., 1999). How this could relate to the prediction of signal peptides in fungal and oomycete effectors remains to be determined.

From the fungal effector set, all secretion predictors, including the best-performing tools SignalP-NN 2, SignalP-NN 3, and TargetP, were consistently unable to predict a signal peptide for only three effectors: Avra10, Avrk1, and Vdlsc1 (Table 4). Similarly for the oomycete effector set, all secretion predictors including the best-performing tools SignalP 2-HMM and SignalP-HMM 3 were unable to predict a signal peptide for only a single oomycete effector (Pslsc1). These four effector proteins have been demonstrated to be secreted via non-classical pathways (Ridout et al., 2006; Liu et al., 2014). This suggests that the most sensitive methods are only likely to fail to predict the secretion of non-classically secreted effectors and that using a union of multiple methods would not necessarily improve sensitivity for this test set. At this stage the computational identification of non-classically secreted effectors remains challenging and these types of effectors require experimental validation of their secretion. In the future, an increased understanding of non-classical secretion mechanisms of fungal and oomycete effectors might lead to improved computational prediction of these effectors. Protein tribe clustering with subsequent examination of high-priority effector candidate families (Saunders et al., 2012) or the presence of conserved protein domains has been effectively applied to identify related effector candidates lacking a predicted signal peptide. However, as the vast majority of fungal effectors share little sequence homology, the utility of this method is limited. Furthermore, orthologs of a secreted protein are not necessarily also secreted (Poppe et al., 2015). Therefore, secretomes predicted through the additional use of reciprocal BLASTs and/or tribe analysis are likely to include a high number of false positives.

**Table 4:** **Fungal and oomycete effectors that were not predicted to be secreted by the prediction tools tested**.

	**SignalP 4.1**	**SignalP-NN 3.0 (*D*-Score)**	**SignalP-HMM 3.0**	**SignalP-NN 2.0**	**Signal-HMM 2.0**	**Phobius**	**TargetP 1.1**
Fungal effectors	Avra10 Avrk1 Vdlsc1 AvrP4 SIX5	Avra10 Avrk1 Vdlsc1	Avra10 Avrk1 Vdlsc1 Avr-Pita2	Avra10 Avrk1 Vdlsc1	Avra10 Avrk1 Vdlsc1 Avr-Pita2	Avra10 Avrk1 Vdlsc1 Avr-Pita2 AvrP123	Avra10 Avrk1 Vdlsc1
Oomycete effectors	Pslsc1 CRN1 CRN2 CRN15 CRN16 CRN63 CRN115 Avr1k ATR5	Pslsc1 CRN1 CRN15 CRN16 ATR5	Pslsc1	Pslsc1 CRN16	Pslsc1	Pslsc1 CRN16	Pslsc1 CRN16 Avr1k ATR5

**Figure 2:**
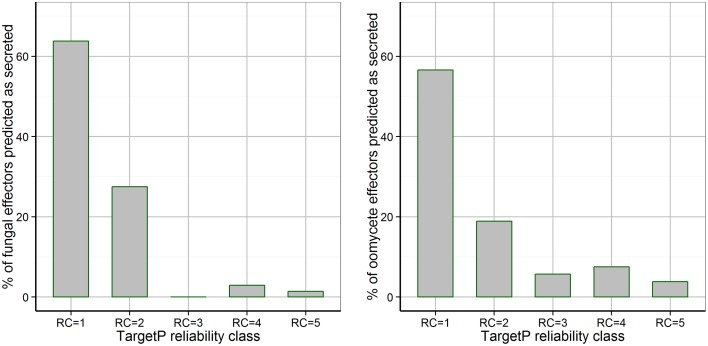
**Distribution of TargetP reliability classes for fungal and oomycete effectors that are predicted to be secreted by TargetP**. The TargetP reliability class distribution for fungal and oomycete effectors is shown, where 1 represents the strongest prediction. The majority of effectors are predicted as secreted with the highest reliability class of 1, however, many effectors are predicted with low reliability classes of 2–5.

TargetP predicted signal peptides with the highest reliability class (RC = 1) for only 63.8% of fungal effectors and for 56.6% of oomycete effectors (Figure 2). Without a restriction on the reliability class (RC from 1 to 5), TargetP predicted “secreted” as the localization for 95.6% of the fungal effectors (three effectors were predicted as “unknown”), whereas it returned “secreted” for 92.4% of the oomycete effectors (two effectors were predicted as “unknown” and two were predicted as “mitochondrial”). Therefore, a restriction on the predicted reliability class should not be used for predicting the secretion of effectors and the exclusion of proteins predicted to be localized to mitochondria has to be used with caution for oomycete effectors.

The relatively poor performance of SignalP 4 for oomycete effectors (Figure 1, sensitivity 83%) is surprising and suggests that previous versions of SignalP (SignalP 2, SignalP 3) should be used for effector mining in oomycete genomes instead. In particular, SignalP 4 does not predict a signal peptide for six out of seven Crinkler effectors in the test set (Table 4; CRN1, CRN2, CRN8, CRN15, CRN16, CRN63, CRN115). Crinkler effectors are a large family of modular proteins that are translocated into host cells, featuring a signal peptide followed by a LXLFLAK sequence motif and C-terminal domains (Haas et al., 2009; Schornack et al., 2010). On the set of seven Crinkler effectors, SignalP 4 achieves the lowest sensitivity, whereas the hidden Markov model predictors of SignalP 2 and SignalP 3 (SignalP-HMM 2, SignalP-HMM 3) correctly predict the signal peptide in all seven Crinklers (Table 4). This exemplifies the substantial benefits of using previous versions of SignalP (SignalP 2, SignalP 3) for oomycete effector mining.

Signal peptide prediction tools such as SignalP return the set of proteins that are likely to carry a signal peptide for the classical pathway, but do not necessarily imply that a protein will be extracellular. Many proteins with a signal peptide are retained in various cellular compartments and thus, signal peptide prediction is often combined with additional evidence for extracellular protein secretion, such as the absence of transmembrane domains, GPI anchors or retention signals (Table 1). We found that no transmembrane regions outside the signal peptide region (first 60 aas) were predicted for any of the 69 fungal effectors using TMHMM or Phobius. For the 53 oomycete effectors, TMHMM and Phobius both return one transmembrane helix outside the signal peptide region for the RXLR effector PITG_03192. This might be an indication that TMHMM and Phobius can be used as a preliminary filter to exclude proteins with multiple, non-N-terminal transmembrane domains for effector mining in fungi. However, these tools should be used with less stringent requirements for effector prediction in oomycetes.

### Subcellular localization prediction tools should not be used for predicting effector secretion

**Figure 3:**
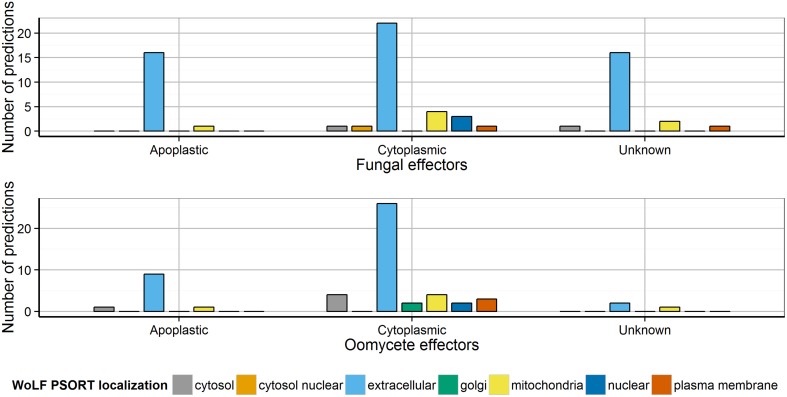
**Distribution of the predicted localization of apoplastic and cytoplasmic effectors using WoLF PSORT**. The distribution of localization predicted by WoLF PSORT is shown for apoplastic and cytoplasmic effectors. Most apoplastic effectors were predicted as extracellular by WoLF PSORT, whereas 34.2% of the cytoplasmic effectors were not predicted to be extracellular.

Prediction of subcellular localization is important for inferring hints about a protein's function. In eukaryotes, a number of compartments exist to which proteins may be localized, e.g., the extracellular space, mitochondria, chloroplast, nucleus, peroxisome, cytosol or plasma membrane. Several plant pathogenomics studies have used the subcellular localization of “extracellular” as a criterion for predicting secretion, commonly using WoLF PSORT which has been trained separately on fungi, animal and plant data. However, we found that applying WoLF PSORT (fungi) to the sets of experimentally verified fungal and oomycete effectors returned 25 cytoplasmic effectors that are not predicted to be extracellular (34.2% of cytoplasmic effectors, Figure 3). This could be explained as follows. First, the estimated sensitivity and specificity of WoLF PSORT is fairly low at around 70% (Horton et al., 2007), which might lead to a high number of false predictions. However, we found that false predictions occurred in particular for non-apoplastic effectors (Figure 3). It is possible that WoLF PSORT may have predicted a signal for host cell localization in effectors rather than for the extracellular secretion of the effector from the pathogen cell. Thus, WoLF PSORT should be used with caution when predicting secretomes and its “extracellular” predictions should not be solely relied upon for effector prediction. An alternative approach is to impose a high level of stringency to WoLF PSORT predictions, as was the case for the *F. graminearum* secretome in which proteins were reported as secreted if the extracellular score was >17 (Brown et al., 2012). Whilst this practice is likely to drastically reduce the number of false positives in the secretome, it is prone to miss *bona fide* effectors that are not predicted to be extracellularly localized. In this study, of the oomycete Crinkler effectors CRN1, CRN2, CRN8, CRN15 and CRN16 which are known to localize to the host cell nucleus (Schornack et al., 2010), WoLF PSORT only predicted a nuclear localization for CRN16. Therefore, the predictions of subcellular localization tools may need to be used with caution in effector prediction studies.

### Practical recommendations for prediction of extracellular proteins in fungi and oomycetes

In this study, we have assessed the performance of various secretion and subcellular localization prediction tools, when applied to datasets derived from known fungal and oomycete effectors, as well as extracellular and intracellular fungal proteins. Based on our benchmarking, we deduce recommendations for extracellular protein prediction in fungal and oomycete pathogen genomes.

We observe that previous versions of SignalP (2, 3) demonstrate increased sensitivity over the latest version (4.1) for predicting signal peptides of oomycete effectors, with the HMM-based methods outperforming the NN-based methods. Indeed, this has formed the basis for the pipeline PexFinder (Phytophthora Extracellular Proteins Finder), which automates identification of oomycete extracellular proteins from EST data (Torto et al., 2003). PexFinder uses SignalP 2.0 but applies an additional logical filter that predicts a protein to be secreted only if both the hidden Markov model predicts a signal peptide and the neural network predicts a cleavage site between amino acids 10 and 40. Whilst this pipeline was proposed over a decade ago, it still retains its value for mining effectors from oomycete genomes.

**Figure 4:**
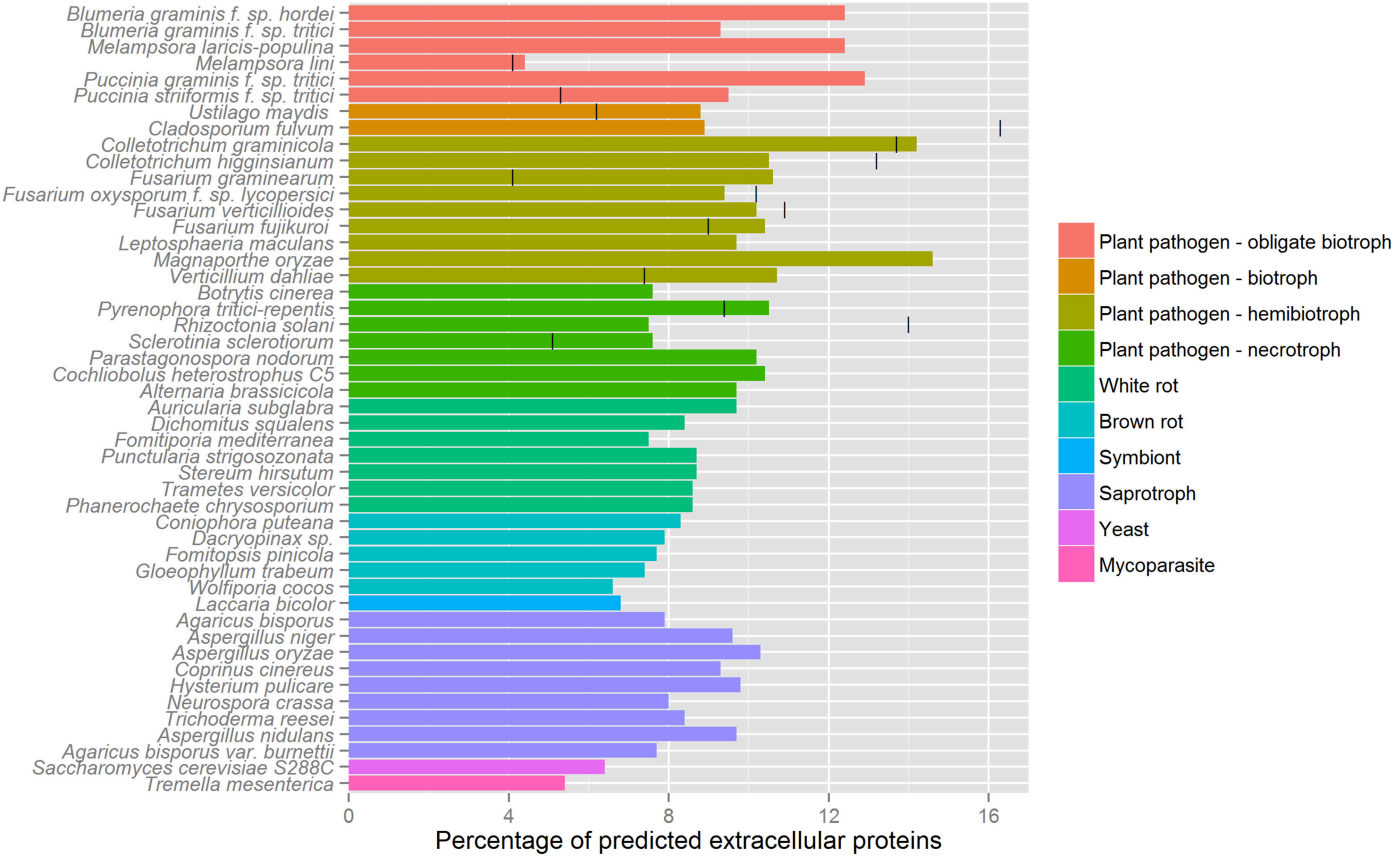
**Predicted secretome sizes in fungi**. The percentages of proteins that are predicted to be secreted are shown for various fungal genomes. Where provided in the literature, previously estimated secretome sizes are indicated with a vertical bar, as given in Table 1. We used the following pipeline for secretome prediction in fungi: SignalP 3.0 *D*-score, a TargetP “secreted” or unknown localization (no restriction on RC score) and no predicted transmembrane domains starting outside the first 60 aas using TMHMM. Genome and secretome size references are given in Table 1, additional genomes used are as follows: *Blumeria graminis* f. sp. *tritici* (Wicker et al., 2013); *Leptosphaeria maculans* (Rouxel et al., 2011); *Magnaporthe oryzae* (Dean et al., 2005); *Botrytis cinerea* (Amselem et al., 2011); *Parastagonospora nodorum* (Hane et al., 2007); *Auricularia subglabra, Dichomitus squalens, Fomitiporia mediterranea, Punctularia strigosozonata, Stereum hirsutum, Trametes versicolor, Coniophora puteana, Dacryopinax sp*., *Fomitopsis pinicola, Gloeophyllum trabeum, Tremella mesenterica, Wolfiporia cocos* (Floudas et al., 2012); *Laccaria bicolor* (Martin et al., 2008); *Agaricus bisporus* (Morin et al., 2012); *Aspergillus niger* (Andersen et al., 2011); *Aspergillus oryzae* (Machida et al., 2005); *Coprinus cinereus* (Stajich et al., 2010); *Alternaria brassicicola, Cochliobolus heterostrophus, Hysterium pulicare* (Ohm et al., 2012); *Neurospora crassa* (Galagan et al., 2003); *Trichoderma reesei* (Martinez et al., 2008); *Agaricus bisporus var. burnettii* (Morin et al., 2012); *Saccharomyces cerevisiae* S288C (Goffeau et al., 1996); *Aspergillus nidulans* (Galagan et al., 2005); *Phanerochaete chrysosporium* (Ohm et al., 2014).

In contrast with oomycete effectors, the NN predictors of SignalP 2 and 3, as well as TargetP, were observed to be the most sensitive for predicting signal peptides of fungal effector proteins. Unlike oomycete effectors, no TM domains were predicted outside the N-terminal signal peptide region using TMHMM or Phobius. Therefore, we propose that for fungal effector mining the requirement of a predicted signal peptide using either SignalP-NN 2 or 3, a TargetP localization prediction of “secreted” or “unknown” (with no restriction on the RC score) and a lack of transmembrane domains outside the signal peptide region (TMHMM/Phobius) would be a robust method. Applying this proposed pipeline to publicly available fungal genomes (some with secretome predictions given in Table 1) highlights the wide variability in the number of predicted secreted proteins produced by the different techniques used in previously published studies (Figure 4). In line with previous reports, we observe a higher percentage of proteins that are predicted to be secreted in pathogens with a biotrophic phase, compared to necrotrophs and saprophytes (Lowe and Howlett, 2012; Lo Presti et al., 2015). By our method, similar numbers of predicted secreted proteins were predicted across multiple species of the same trophic class, whereas reported numbers were highly variable in genome survey publications for these species (Figure 4).

## Conclusion

Prediction of effector proteins is of vital importance to the field of plant pathology, and relies heavily on the strengths or weaknesses of secretion prediction software. In this study, we assess the performance of popular software tools against known effectors of both the fungi and oomycetes and offer recommendations on which may be better suited to specialized applications. However, such performance evaluations inevitably vary based on the test data sets used, and therefore, we advise readers to carefully consider the suitability of these recommendations to their own data. Based on the results discussed herein, we recommend the use of the neural network predictors of SignalP 2 or 3, a TargetP localization prediction of “secreted” as well as transmembrane protein removal using either TMHMM or Phobius as a robust choice for predicting the secretion of fungal effectors. In comparison, the hidden Markov model predictors of SignalP 2 and 3 perform best for predicting the signal peptide of oomycete effectors and automated pipelines such as PexFinder retain their value (Torto et al., 2003). However, the secretome includes many proteins unrelated to pathogenicity, and a number of additional conditions must be subsequently assessed in order to arrive at a subset that represents a potential set of effectors. In oomycetes, this can be achieved using motif enrichment analysis based on RXLR or Crinkler effector families (Petre and Kamoun, 2014), whereas in fungi this process is not feasible and alternative criteria such as small size, an enrichment in cysteines, genomic location, or signatures of diversifying selection can be used (Sperschneider et al., 2015).

While the reliability of secretion prediction is highly relevant to effector prediction, one must not overlook the potential for errors to arise from prior steps involved in the generation of sequence resources. The annotation of gene structure in effector genes can be particularly error-prone for various reasons, stemming from idiosyncrasies related to their genomic context for example their tendency to be associated with repetitive regions of the genome (Raffaele and Kamoun, 2012). There is potential for errors to occur in the assembled genome sequence, especially for those assembled from short-read data only, and the subsequent use of automated annotation pipelines can contribute to inaccurate or fragmented gene predictions. If this occurs in the 5′ region it can lead to misprediction of N-terminal signal peptides. We also note that due to high gene density in fungi that transcript UTRs of adjacent gene loci frequently overlap (Guida et al., 2011; Wang et al., 2014), potentially resulting in gene annotations that are merged products of two or more adjacent loci. Therefore, the use of RNA-seq-based annotation methods specifically designed for fungi (Reid et al., 2014; Testa et al., 2015) can be beneficial to arrive at an optimal set of gene annotations for subsequent secretion prediction.

Our results showed that one of the areas that is currently suffering from poor accuracy is the prediction of subcellular localization for effector proteins that are first secreted from the fungus and then targeted to a host organelle. In particular, we recommend that the requirement of extracellular localization as predicted by WoLF PSORT should not be used for effector mining in secretomes. Re-training subcellular localization tools with updated data sets including experimentally validated effectors might help to improve accuracy. There are few well-studied fungal effectors with confirmed host-localization, one being the SP7 effector of the arbuscular mycorrhiza *Glomus intraradices* (Kloppholz et al., 2011). SP7 is initially secreted to the apoplast, then imported into the host cell, and then into its nucleus. This localization is determined by multiple motifs, including a signal peptide, nuclear localization domain and an array of imperfect tandem hydrophilic repeats possibly involved in membrane integration. Both TargetP and WoLF PSORT predicted that the complete version of SP7 was secreted, however after removal of the signal peptide based on SignalP analysis, the TargetP prediction changed to “other” and WoLF PSORT (plant mode) predicted nuclear localization. Intriguingly, this suggests that subcellular localization prediction has the potential to become a powerful tool for providing insight into potential modes of action for candidate effectors based on their organelle targets. Additionally, there are currently no tools designed to predict proteins secreted in a non-classical manner that have been specifically trained on either fungi or oomycetes sequences due to a lack of training data. Although tools like SecretomeP are able to predict some cases (Liu et al., 2014), in the future refined tools for non-classical secretion prediction could be a source of significant improvements in effector prediction.

In summary, whilst existing methods for signal peptide prediction achieve high accuracy, the main areas for improving eukaryotic effector secretion prediction will come from advances in subcellular localization prediction tools as well as from investigations of non-classical secretion pathways and improved gene prediction tools for pathogen genomes.

## Author contributions

JS conceived the study and all authors contributed to the design of the study. JS, AW, and JH acquired, analyzed and interpreted the data. All authors drafted the manuscript and approved the final version.

## Funding

JS was partially supported by the Australian Grains Research and Development Corporation.

### Conflict of interest statement

The authors declare that the research was conducted in the absence of any commercial or financial relationships that could be construed as a potential conflict of interest.
